# Outcomes and Critical Factors for Successful Implementation of Organizational Health Literacy Interventions: A Scoping Review

**DOI:** 10.3390/ijerph182211906

**Published:** 2021-11-12

**Authors:** Marise S. Kaper, Jane Sixsmith, Sijmen A. Reijneveld, Andrea F. de Winter

**Affiliations:** 1 Department of Health Sciences, University Medical Center Groningen, University of Groningen, Hanzeplein 1, P.O. Box 30.001, FA10, 9700 RB Groningen, The Netherlands; s.a.reijneveld@umcg.nl (S.A.R.); a.f.de.winter@umcg.nl (A.F.d.W.); 2 Center for Dentistry and Oral Hygiene, University Medical Center Groningen, University of Groningen, Antonius Deusinglaan 1, 9713 AV Groningen, The Netherlands; 3 Health Promotion Research Centre, National University of Ireland Galway, University Road, H91 TK33 Galway, Ireland; jane.sixsmith@nuigalway.ie

**Keywords:** health literacy, organization and administration, health care settings, organizational innovation, culture, program development

## Abstract

Organizational health literacy (OHL)-interventions can reduce inequality and demands in health care encountered by patients. However, an overview of their impact and critical factors for organization-wide implementation is lacking. The aim of this scoping review is to summarize the evidence on: (1) the outcomes of OHL-interventions at patient, professional and organizational levels; and (2) the factors and strategies that affect implementation and outcomes of OHL-interventions. We reviewed empirical studies following the five-stage framework of Arksey and O’Malley. The databases Scopus, PubMed, PsychInfo and CINAHL were searched from 1 January 2010 to 31 December 2019, focusing on OHL-interventions using terms related to “health literacy”, “health care organization” and “intervention characteristics”. After a full-text review, we selected 24 descriptive stu-dies. Of these, 23 studies reported health literacy problems in relation to OHL-assessment tools. Nine out of thirteen studies reported that the use of interventions resulted in positive changes on OHL-domains regarding comprehensible communication, professionals’ competencies and practices, and strategic organizational changes. Organization-wide OHL-interventions resulted in some improvement of patient outcomes but evidence was scarce. Critical factors for organization-wide implementation of OHL-interventions were leadership support, top-down and bottom-up approaches, a change champion, and staff commitment. Organization-wide interventions lead to more positive change on OHL-domains, but evidence regarding OHL-outcomes needs strengthening.

## 1. Introduction

Almost one in every two people in Europe encounter problems handling health issues because of limited health literacy skills [1]. These problems are more prominent among people of a higher age and lower educational level [1]. Health literacy is defined as ‘the degree to which people are able to access, understand, appraise and communicate information to engage with the demands of different health contexts’ [2]. As Rudd et al. consistently point out [3,4,5], a health literacy gap is emerging between the abilities of patients and the demands placed by increasingly complex health services. This gap can contribute to a range of negative consequences for people with limited health literacy [1,6], who find it difficult to access and navigate health care organizations, communicate with health professionals, understand information, and engage in decision making and self-management [3,6,7,8,9,10]. These consequences can have a profound impact on patients, affecting their safety, quality of care, and health outcomes [1,6]. In order to reduce and prevent these problems, it has been recommended to reduce the complex demands in health care organizations [5,11,12,13].

Health care organizations can reduce these demands and “make it easier for people to navigate, understand, and use information and services to take care of their health” [13,14], which is the definition of the concept of organizational health literacy (OHL). Contextualising health literacy to health care organizations involves the design of accessible and easy to use health services, including health promotion and ill health prevention, fostering equality and a responsive health system, supporting people to navigate that system, and engaging them in making informed health related decisions [15,16,17]. Palumbo [15] conducted a literature review with a preventive medicine orientation, and distinguished five themes from the identified OHL literature: (1) understanding OHL as a preventive health policy issue and promoting the integration of health literacy into organizations po-licies and daily activities; (2) contextualizing OHL in a patient-centred care perspective, building on a combination of formal (top-down) and informal (bottom-up) approaches to improve the accessibility of health services and engagement of patients; (3) raising awareness and strengthening commitment for achieving OHL, (4) preparing a health literate workforce using tailored training and capacity building, and (5) measuring efforts and outcomes related to OHL by using a systematic approach. Measurement should focus on the ability of health organizations to engage patients in a co-creation relationship, the quality of communication, supportive services and technologies.

Reducing the organizational demands for people with limited health literacy requires a combination of approaches targeted at the level of patients, professionals and the organization [13,18], denoted as organizational health literacy (OHL)-interventions. At the patient level, interventions can improve oral, written, and digital communication, and accessibility of services and physical navigation, as well as involve patients more actively in improving health information and services. At the professional level, OHL-interventions can improve capacity building and promotion of health literacy friendly communication practices. OHL improvement at the organizational level involves domains such as leadership and culture, organizational policies, systems processes, and structures. Over the last decade, a number of such OHL-interventions have been developed [19,20]. These interventions usually involve two phases: (1) assessment of health literacy problems from the perspectives of patients, professionals and independent observers; and (2) planning and application of interventions aimed at reducing demands in healthcare organizations. 

Two reviews concluded that evidence on the planning, application, and outcomes of OHL-interventions was limited [19,20]. Until recently, these interventions focused mostly on the assessment of health literacy problems at the patient and professional levels, including physical navigation, and written-, digital-, and spoken communication, but with limited attention to an organization-wide approach [19,20]. The available studies of applied interventions reported a number of facilitators and barriers that influenced OHL-interventions, such as lack of health literacy awareness, staff commitment, and leadership support [19,20]. The evidence on outcomes indicated that implementation periods were brief and improvement of OHL-outcomes limited. 

Since the publication of these reviews, new insight has been gained regarding outcomes and implementation of OHL-interventions, and on how organizational transformation may improve patient outcomes. Current research on health literate organizations focuses more on facilitating sustainable transformation to improve OHL outcomes at patient, professional and organizational levels [14]. This scoping review summarizes the evidence regarding: (1) outcomes of OHL-interventions at patient, professional and organizational levels; and (2) factors and strategies that influence implementation and outcomes of these interventions. 

## 2. Materials and Methods

To guide this scoping review we used the five-stage framework for scoping reviews developed by Arksey and O’Malley (2005) [21]. The five stages are: (1) Identify the research questions, (2) Identify and retrieve relevant articles, (3) Select articles, (4) Chart the data, (5) Collate, summarize and report. We structured the methods section in line with these stages.

### 2.1. Stage 1. Identify the Research Questions 

Before conducting the review, within the group of authors we defined two preliminary research objectives and discussed the concepts to guide the literature search. We aimed at a sensitive search to catch all potentially relevant studies regarding the domains of OHL interventions, and criteria to specify the interventions regarding the phases of assessment and application of OHL interventions.

### 2.2. Stage 2. Identify and Retrieve Relevant Articles

First, to identify and retrieve relevant articles, we set up a literature search strategy based on search terms and inclusion criteria used in two previous reviews of OHL interventions [19,20]. Second, with the help of a librarian (TvI), we refined the research objectives and search strategy, and developed a protocol, all of which we discussed among the co-authors (MK, JS, SAR, AFdW). This was to ensure that methods and search strategies were consistent and comprehensive. We applied the final search strategy to the MEDLINE/PubMed databases and then adapted it for the other databases, covering all publications up to 31 December 2019. We searched the databases PubMed, Scopus, PsychInfo and CINAHL. In the literature search we included keywords and MESH terms related to the concept of “health literacy”; we combined these with Boolean operator AND search terms related to the health care setting, and Boolean operator OR search terms involving intervention characteristics. The complete search string is provided in Table S1 as supplementary material.

To ensure inclusion of all relevant studies in the review we used reference searches of retrieved articles to complement the electronic searches. Inclusion criteria were: (1) publication between January 2010 and December 2019; (2) inclusion of an abstract written in English; (3) an OECD country as geographical setting; (4) a study setting involving a health care setting in primary or secondary care; (5) a study aimed at assessment of organizational barriers and improvement of outcomes for adults with limited health lite-racy; (6) a study design involving an intervention, evaluation of a program, a pilot-study or needs assessment; (7) an intervention focused on assessing problems or changing two or more domains of organizational health literacy: changes at patient level (oral, written and digital communication and health literacy levels); changes at professional level (health literacy capacities and communication practices); or changes at organizational level (leadership and culture, organizational policies, systems processes, and structures).

### 2.3. Stage 3. Selection of Articles

After removing duplicate articles, we reviewed the title and abstract of identified articles against the following exclusion criteria: (1) health literacy was assessed or addressed only at the individual or family level (e.g., validation of screening tools or educational interventions for patients); (2) the only focus was to investigate determinants associated with health literacy and health outcomes; (3) the aim was to develop and validate instruments to measure organizational health literacy without investigating their implementation in organizations.

One investigator (MK) did the initial screening. In cases of uncertainty, a second investigator (AFdeW) reviewed the abstract or full text of an article; together consensus was reached on inclusion or exclusion in the review. Articles identified for inclusion underwent full text screening and two investigators screened a sub-section to ensure fit to criteria and consistency. 

### 2.4. Stage 4. Charting the Data

In three steps we extracted the data from the selected studies in Excel, sorted them in tables, and analysed them based on the study purpose. First we extracted descriptive data: author, year and country, design and evaluation method, aim, setting, sample, and OHL-intervention components. Second, we extracted data on outcomes of OHL-interventions at patient, professional and organizational levels. Third, we extracted data on whether critical factors and strategies were considered to be facilitators or barriers to implementation processes. 

### 2.5. Stage 5. Collate, Summarize and Report

In three steps we extracted the data from the selected studies, sorted them in tables and analysed them based on key themes informed by the study purpose, to: (1) assess the outcomes of OHL-interventions, and (2) to unravel the factors and strategies affecting the implementation and outcomes of OHL-interventions. First, we tabulated the selected studies by author, year and country, research design, setting, sample, OHL domains addressed, and focus of the study, i.e., assessment or application of OHL-interventions. Second, we summarized and reported the outcomes of OHL-interventions following their assessment or application, and the level to which the outcome applied: patient, professional, and/or organization. Third, we summarized and reported factors and strategies which influenced the assessment and application of OHL-interventions, and analysed whether these were facilitators or barriers at patient, professional, and/or organizational level. 

## 3. Results

**Figure 1:**
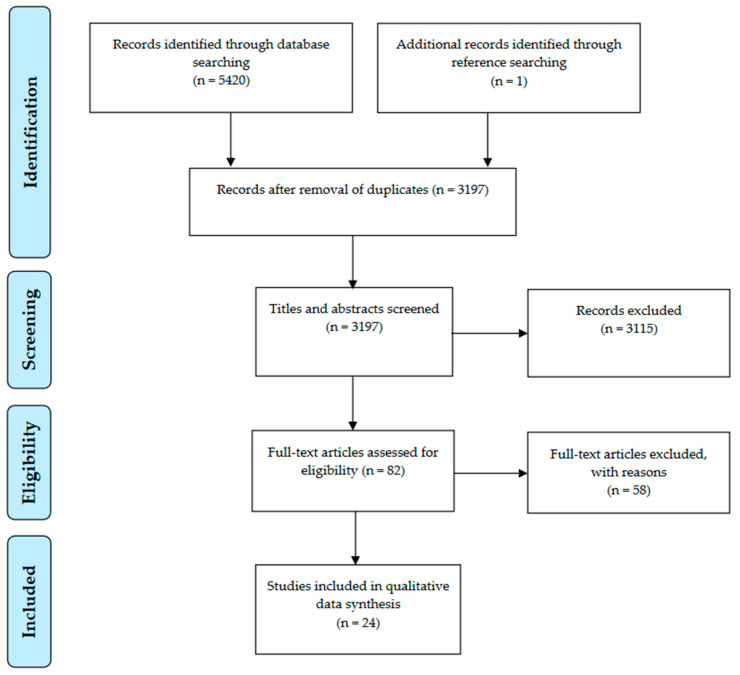
Flow of studies through the review.

We identified 5420 records from the literature search and one record through reference searching (we retrieved 1511 records from Pubmed; 1351 from Scopus; 1750 from Cinahl; and 808 from Psychinfo). After removing 2223 duplicates, we screened 3197 titles and abstracts and included 82 articles for full-text review. After reading the full text, we selected and excluded articles based on the criteria specified above. We included twenty-four articles in the data extraction. This results section presents: (1) description of the studies, (2) outcomes of OHL-interventions, and (3) strategies and factors that influence the implementation of OHL-interventions. Figure 1 presents the results of the literature search and study selection. 

### 3.1. Description of Studies

The 24 selected articles involved 17 original research projects (Table 1); several studies were part of larger research projects (these were: Grabeel [22], Grabeel [23], and Tester [24]; Beauchamp [25], Goeman [26], and Jessup [27]; Mabachi [28] and Brega [29]; Vellar [30] and Mastroianni; Weaver [31] and Wray [32]). We included some articles because, although they reported on a single domain, they were connected with other articles reporting different domains of the same study. We sorted the studies according to the results of the assessment of OHL-domains, and the planning and delivery of interventions aimed at improvement of health literacy related problems. Unlike the study conducted by Cawthon et al. [33], the remaining 23 studies conducted an OHL-assessment. Thirteen of these studies focused solely on assessment of health literacy related problems [3,17,22,23,24,34,35,36,37,38,39,40]. Together with the assessment, these studies also often evaluated the feasibility of the OHL-instrument. Eleven studies reported on both the assessment and on findings regarding the planning and delivery of interventions [22,25,26,27,28,29,30,31,32,33,41,42,43]. Fourteen studies were conducted in the United States [22,23,24,28,29,31,32,33,34,36,38,39,40,43]; other studies were conducted in Australia [25,26,27,30,41], New Zealand & Canada [37], and several European countries including Austria [35], Italy [17], Ireland and the Netherlands [42], and Spain [3]. Study settings involved hospitals, as well as general health care settings like community and primary care practices, pharmacies and dental clinics. 

The majority of the studies used a mixed-method approach (n = 16), or qualitative (n = 4) or quantitative approaches (n = 4). Multiple informants and methods were used to report on the assessment and application of OHL-interventions; these included managers, professionals, patients and observers who had taken part in surveys, interviews, focus group discussions, and observation and review of documents. The interventions targeted a variety of OHL domains using different tools and approaches. Domains most frequently addressed were the comprehensibility of written patient information materials, digital communication, oral communication, and navigation. Fewer studies targeted OHL as a strategic priority, health literacy policies, and capacity building of staff [17,25,30,31,32,35]. A number of studies [3,17,31,36,40,42] used or adapted the toolkit “The Health Literacy Environment of Hospitals and Health Centers. Partners for Action: Making Your Healthcare Facility Literacy-Friendly” (HLEHHC Toolkit) developed by Rudd and Anderson [44]. Other studies used, e.g., the HLUP toolkit [28,29,34] or the Agency for Healthcare Research and Quality (AHRQ) Health Literacy Assessment Tool [39,43]. 

### 3.2. Outcomes of OHL-Interventions

In this section, we present first the outcomes of the OHL-assessments, and second the impact after the delivery of interventions (Table 2). Findings are the result of descriptive studies. Most studies (n = 23) assessed and identified OHL-related problems at the levels of patients, professionals and organizations. Patients encountered problems rela-ting to navigation, spoken communication, and understanding and acting upon written and digital information [3,17,23,25,28,29,30,31,34,36,37,38,39,40,41,42,43], although they also reported positive experiences [3,31,36,40]. Professionals reported limited understanding of health literacy, a lack of training, and infrequent use of recommended health literacy practices, such as use of plain language and the teach-back method [3,17,24,25,26,27,30,31,34,35,36,37,38,39,40,42,45]. Other studies reported that professionals had a patient-centred attitude and applied health li-teracy practices, but on an informal basis [17,36]. However, the assessment itself often increased awareness of health literacy problems among professionals [3,30,31,34,42]. At the organizational level, OHL was rarely considered a strategic priority, and strategic plans, policies, and routine procedures were often considered insufficient to address pro-blems related to OHL [17,25,30,31,35,38,42,43]. For example, the concept of patient-centred care was not translated into a concrete plan, and procedures to improve coordination of care were lacking [3,17,37,38].

The application of organization-wide OHL-interventions resulted in some improvement of patient outcomes [25,26,27,30,41], and greater changes in intermediate outcomes at professional and organizational levels [25,26,27,30,32,33,41,42]. Despite relatively small sample sizes, two research projects reported some improvement in patient-related outcomes [25,26,27,30,41], such as increased health literacy skills, participation in health care, and increased self-management abilities following interventions involving peer community members. Although not evaluated by patients, independent assessors reported both improved comprehensibility related to patient information materials [30,41], and some li-mited changes in the complexity of materials [29]. Improved health outcomes were not reported. Studies which reported greater change on intermediate outcomes at professional and organizational levels [25,26,27,30,32,33,41,42] used an organization-wide and long-term approach to deliver OHL-interventions. After training, (health) professionals in these studies reported increased competency to address health literacy and application of recommended practices [25,26,27,30,32,41,42]. Intermediate outcomes at the organizational level included integration of OHL into policies and systems, redesign of services, organization-wide programs to promote staff capacity building, and promotion of health literacy strategies by professionals in written, digital, and spoken communication [25,26,27,30,32,41]. Limited impact was reported regarding routine organization-wide application of practices [25,32,42], navigation, and distal outcomes such as health indicators, quality of care, patient safety, and cost-effectiveness [25,28,29,32,42,43]. A few studies with only brief implementation periods struggled with defining priorities and action plans, and reported limited changes among professionals and organizations [28,29,43], although they undertook preliminary attempts to improve written communication and train staff.

### 3.3. Factors and Strategies Influencing the Application of OHL-Interventions 

Reported facilitators of a comprehensive OHL-assessment were: patient engagement, a change champion, commitment and capacity of staff, support from leadership and researchers, and an innovation culture, see Table 3 [3,28,31,34,39,42,43]. Patient engagement was found to be crucial for identifying health literacy problems from their perspective [25,30,42]. Health professionals needed to perceive the OHL-assessment as relevant and feasible, be committed to its implementation, and have knowledge of quality improvement [3,28,34,42,43]. Clear introduction meetings were found to increase HL awareness and staff buy-in [34,39,42,43]. Support from researchers added credibility to the intervention and promoted its quality of implementation [3,28,34,42,43]. Facilitators at the orga-nizational level were: an innovation culture focused on quality improvement, leadership support, and coordination by a change champion [28,30,32,34,43]. 

Critical facilitators regarding the delivery of OHL-interventions were reported to be: leadership support, an organization-wide approach, a change champion and project committee, sufficient resources, professional commitment and competencies, and patient engagement, in order to achieve improvement at professional and organizational levels, see Table 3 [25,26,27,30,31,32,33,41,42]. An organization-wide approach, supported by senior ma-nagement, was reported to stimulate the development of program logic models, strategic prioritization, and planning of OHL improvement [25,30,32]. These organizations often reported having simultaneously used top-down and bottom-up strategies to increase staff commitment to and knowledge of change strategies and quality improvement [25,30,32]. Co-design strategies and PDCA cycles were applied to develop, refine, and test interventions [25,30,32]. In contrast to the assessment phase, patients seemed to be less engaged in the application of interventions [25,42]. Only in the studies of Vellar et al. (2017) and Mastroianni et al. (2019) [30,41] were patients systematically involved in processes to improve navigation and patient-information materials. In the research project of Beauchamp et al. (2018) [25], small samples of patients were involved in the development and testing of interventions. Studies that found OHL-interventions to have only a limited impact reported that their implementation periods were brief, and affected by barriers such as lack of a change champion and coordinated planning processes [29,43], as well as limited time, resources and leadership support [22,28,29,43]. 

## 4. Discussion

The aim of this scoping review was to summarize the evidence regarding: (1) outcomes of OHL-interventions at patient, professional and organizational levels; (2) factors and strategies that influence the implementation and outcomes of OHL-interventions. We selected 24 articles, which included 17 original research projects (fully) based on qualitative and quantitative descriptive studies. With regard to the outcomes we: (a) identified OHL-related problems across patient-, professional- and organizational levels [3,25,32,34,36,37,38,42]; and (b) found that application of organization-wide OHL-interventions resulted in some improvement of patient outcomes [25,26,27,30,31,32,41], and greater change in intermediate outcomes at professional and organizational levels [25,26,27,30,32,33,41,42]. However, some studies reported only limited change [28,29,43], and no studies reported improvement on more distal outcomes. We found that several critical factors and strategies facilitated organization-wide outcomes of OHL [25,26,27,30,31,32,33,41,42]: leadership support, an organization-wide approach, an innovation culture, a change champion, commitment and adequate capacity of staff, and patient engagement. 

Compared with the earlier reviews of Farmanova et al. [19] and Lloyd et al. (2018) [20], our findings confirmed the evidence regarding identified OHL-related problems, and we observed greater progress on the impact of organization-wide OHL interventions [25,26,27,30,31,32,33,41,42]. A first point regarding our evidence is that the number of OHL-pro-blems identified across a variety of countries underlines the need to use comprehensive frameworks to improve organizational health literacy in health care settings [14,35,46,47,48,49,50].The progress we observed related particularly to recent studies, which showed how a single health literacy project led to development of a health literate organization by employing a systematic and organization-wide approach. These studies strengthened the evidence particularly on three points: (1) patient outcomes showed some evidence of increased health literacy, understanding of information, and participation in health care [25,26,27,30,41]; (2) outcomes among health professionals showed evidence of improved competencies and practices to address health literacy [25,30,32,42]; (3) intermediate organizational outcomes showed evidence of embedding of OHL into policies and structures, staff training, and interventions to improve screening, communication and patient engagement [25,26,27,30,31,32,33,41,42]. This review thus indicates a growing awareness of how to achieve sustainable improvement on various OHL-domains, and supports the findings in recent reviews by Zanobini et al. (2020) [18] and Meggetto et al. (2020) [51]. 

Our review points to several critical facilitators and strategies that can promote health literacy friendly organizations in the long term: leadership support, an organization-wide approach, an innovation culture, a change champion, commitment and capacity of staff, and patient engagement [25,26,27,30,31,32,33,41,42]. These facilitators correspond with findings reported in other studies on innovation in health care settings [52,53,54,55,56] and universal processes for organizational change [19,20]. In our review, some studies reported limited outcomes because they had a shorter duration (six months) [28,29,43], struggled with coordination, staff turnover, and a lack of a change champion as well as leadership support and resources [28,29,43]. Other studies in our review suggest that a systematic organization-wide approach is more promising [25,26,27,30,31,32,33,41,42]. These implementation strategies involved simultaneous use of top-down and bottom-up strategies to engage staff and patients; such strategies have been widely used in the field of health promotion [32,57]. This observation underlines the frameworks of Trezona (2017) [47] and Zanobini (2020) [18] in the sense that various OHL-domains are interconnected and need to be targeted simultaneously in order to initiate a cyclical and widening process of improving the quality of health care by making organizations responsive to health literacy [51]. These findings have thus strengthened the evidence base for implementation of OHL-interventions. 

However, our review also shows the evidence for OHL-interventions still to be ge-nerally weak, particularly regarding their effects on more distal outcomes like improved health or cost-effectiveness [18,20]. The first, general, issue regards the total lack of studies with an experimental design: studies conducted only baseline measurements, or had small samples when investigating change over time, and did not compare outcomes with control settings. Second, the instruments for measuring OHL outcomes did not include information on reliability and validity, although some instruments [34,44] indicated ha- ving face validity, and were used in different settings and countries [20]. Recently, several instruments were designed to assess a wide spectrum of OHL-domains [34,44,46,47], and one of these was reported to have satisfactory reliability and validity [49,58]. Although these instruments did not evaluate the outcomes of interventions, they may have the potential to be used for benchmarking and for investigating change over time [49]. 

The particular weakness of the evidence for OHL-interventions is that their impact is still unclear regarding more distal outcomes like patient health outcomes, quality of care, and cost reduction. This may be explained by several factors. First, in our review, mea-surement of more distal outcomes among larger samples of patients was lacking. However, we noted that, in some studies, small groups of patients were engaged in the development and evaluation of interventions [25,30,41], which resulted in improvement of health literacy levels, and in understanding and self-management of patients. Second, it seems plausible that the impact of organization-wide OHL interventions results first in intermediate outcomes among professionals and organizations, outcomes which may be influenced by many factors [14]. Zanobini [18] for example reports that (single) interventions directly targeted at patients result in improved outcomes in patient satisfaction, knowledge, and skills. In sum, promising outcomes may result from studies that combine patient-targeted interventions with systematic approaches directed at professional and organizational levels, and include measurement of distant patient outcomes, quality of care, and cost-effectiveness. 

### 4.1. Strengths and Limitations

Several strengths of this study can be noted. We conducted a comprehensive search strategy and selection procedure to include relevant studies in the review. The fact that the selected studies were conducted in various health care organizations and countries is promising for the generalizability of the results. However, several limitations should be mentioned. First, the approach of a scoping review did not include a quality assessment of the selected studies; this limited the potential to connect content and quality. Second, we focused on peer-reviewed articles which had abstracts in English; this may have led to missing relevant studies from the grey literature or studies published in other languages. We are, however, confident that we have selected the most relevant ones. A final limitation is that publication bias may have influenced this review: studies reporting negative results could be difficult to get published. However, we identified several studies which explicitly reported the problems encountered, and consider the influence of publication bias to be limited. 

### 4.2. Implications

Organization-wide implementation of OHL-interventions can improve intermediate outcomes among professionals and organizations, and has the potential to mitigate health literacy problems among patients. We recommend: (1) assessing OHL problems using a comprehensive and valid instrument; (2) starting with implementation of easy-to-achieve interventions; (3) using a systematic approach to achieve greater organizational change, simultaneously applying bottom-up and top-down approaches; (4) taking into account the critical facilitators of implementation: a change champion vs a project committee, lea-dership support, sufficient resources, patient involvement, and competent and committed staff.

In order to strengthen evidence on OHL-interventions, we need studies with a more rigorous design to evaluate their effectiveness, and which use OHL-instruments that have adequate reliability and validity and are suitable for the European context [14,18,20]. Furthermore, more distal patient-related outcomes like quality of care, safety, and cost-effectiveness should be evaluated. 

Health care organizations have primarily focused on treatment, but there is an increasing recognition of their role in health promotion and prevention in order to address health inequalities in the broader social context [14,15,25]. OHL-interventions are one approach to improve outcomes for individuals with limited health literacy. Other effective strategies may be school-based health literacy education, mass-media communication or empowering individual people as well as communities, and building health literacy competencies of (future) health professionals [59]. As such, OHL-interventions are probably most effective in combination with these other approaches, but this evidently requires further study.

A contextual factor that must be acknowledged in relation to this scoping review is that the period of the literature search preceded the start of the COVID-19 pandemic. The importance of health literacy came to the fore during the COVID-19 pandemic, as the resilience of communities and the relationship of citizens to health care providers depend on it, particularly in crisis situations. This underlines the relevance of this scoping review on OHL-interventions. The COVID-19 pandemic is likely to have influenced the field of OHL-intervention research as health care organizations have, to a greater or lesser extent, faced several periods of crisis due to exceptional service demands. The nature of this influence is unknown. Therefore, we recommend that future studies investigate the influence of the COVID-19 pandemic on the research related to organizational health literacy. Organization-wide OHL-interventions have previously required longer time periods, of several years, for changes to be implemented successfully and sustained. Since the onset of the pandemic in March 2020, health care organizations may have responded in one of two ways: putting the implementation of OHL-interventions on hold or embracing OHL quickly in response to the situation. The COVID-19 pandemic has shown that health settings can accelerate innovation, but whether this holds for OHL-interventions is to be determined.

## 5. Conclusions

Delivery of organization-wide OHL-interventions resulted in some improvement in patient-related outcomes and changes at the professional and organizational levels and may be a promising approach to mitigate health literacy problems. Critical success factors for organization-wide implementation are leadership support, simultaneous top-down and bottom-up approaches, a change champion and project committee, and staff commitment. Efforts to implement organization-wide OHL-interventions should take into account these critical success factors. Organization-wide interventions were reported to achieve more positive change on OHL-domains, but evidence regarding OHL-outcomes needs strengthening. 

## Figures and Tables

**Table 1:** Descriptive results of OHL-interventions regarding research design, aim, setting, sample, and OHL-intervention.

Author, Year	Research Design	Focus	Setting	Sample	OHL-Intervention
De Walt (2011) [34]	Qualitative study: Interviews	Assessment	Primary care practices (n = 10)	Staff and health professionals (number not reported)	Assessment (using HLUP toolkit): 20 tools organized under five sections: - path to improvement - improve spoken communication - improve written communication - improve self-management and empowerment - improve supportive systems
Dietscher (2016) [35]	Mixed methods: Survey Interviews	Assessment	Hospitals (n = 9)	Coordinators (n = 9) Other hospital staff (number not reported)	Assessment (using WGKKO-I toolkit, which has 9 OHL standards) (summarized here): - policy, organizational structures and resources on OHL - staff training and promoting of HL communication - initiation of HL improvement and supportive physical environment - participation of patients in design of services and materials
Grabeel (2018) [22]	Quantitative study: Survey	Assessment	University medical centre	Nurses and other staff (n = 196)	Assessment (using HLEHHC toolkit) of: - current health literacy knowledge - interest in training
Grabeel (2018) [23]	Quantitative study: Rating of materials	Assessment	University medical centre	Sample: NA	Assessment (using HLEHHC toolkit) of printed patient education materials, comparing: - hand-scored SMOG method - computerized F-K grade level method
Tester (2019) [24]	Quantitative study: Structured interviews Observation	Assessment	University medical centre	Patients (n = 298) Observers/auditors	Assessment (using HLEHHC toolkit) of oral communication: - Patient Satisfaction Survey Interview Form (PSSIF) - Oral Exchange Rating Form (OERF)
Groene (2011) [3]	Mixed methods: Surveys Interviews Observation	Assessment	Hospitals (n = 10)	Patients (n = 313) Coordinators (n = 6)	Assessment (using HLEHHC toolkit) of three domains: - navigation: walking interviews undertaken by researcher - written communication: Flesch–Szigriszt readability formula - patients’ perceptions of written and oral communication
Horowitz (2014) [36]	Mixed methods: Surveys Interviews Observation	Assessment	Community-based dental clinics (n = 26)	Dental providers (n = 60) Patients (n = 67)	Assessment (informed by HLEHHC and HLUP toolkits) on four domains: - review of accessibility, signage and navigation, including website and phone - written communication; educational materials and patient forms - provider perspective regarding health literacy friendly communication - patient perspectives regarding navigation, communication and treatment
Lambert (2014) [37]	Qualitative study: Interviews Focus group	Assessment	Primary health care services (n = 4)	Health professionals (n = 29)	Assessment on three domains: - understanding of health literacy and needs of indigenous patients - suitability of the health care environment for people with limited health literacy - opinions and strategies to address health literacy problems
Martinez-Donate (2013) [38]	Mixed methods: Interviews Surveys	Assessment	Clinics provide outreach oncology services (n = 5)	Various clinical staff (n = 41) Patients (n = 53)	Assessment (informed by Chronic Care model) on four domains: - community resources - self-management support - delivery system design - decision support
O’Neal (2013) [39]	Mixed methods (post-test control group): Survey Interviews Observation	Assessment	Community pharmacies (n = 8)	Staff (n = 21) Patients (n = 60) Auditors (n = 4)	Assessment (using AHRQ Health Literacy Assessment Tool) on three domains: - promotion of services and pharmacy environment - printed materials - health literacy-sensitive verbal communication. Brief training intervention on HL knowledge and HL sensitive communication
Shoemaker (2013) [43]	Mixed methods: Document review Observation Interviews	Assessment and delivery	Pharmacies (n = 8)	Coordinating staff (n = 8) Other staff (number not reported)	Assessment (using AHRQ Health Literacy Assessment Tool) on three domains: - promotion of services and pharmacy environment - printed materials - health literacy-sensitive verbal communication
Palumbo (2017) [17]	Mixed methods: Document review Interviews Survey	Assessment	Public hospitals (n = 3).	Senior managers and health professionals (n = 6) Patients (n = 9)	Assessment (using Italian version of HLEHHC toolkit) on five domains: - navigation - printed communication - oral exchange - technology - policy and protocols
Smith (2010) [40]	Mixed methods: Interviews Observation Rating of materials	Assessment	Stroke unit and a senior independent living facility.	Auditors (n = 12) Health professionals and various staff (number not reported)	Assessment (using HLEHHC toolkit) on five domains: - navigation - printed communication—Fry Readability Graph (Schrock, 2009) - oral exchange - technology - policy and protocols
Beauchamp (2017) [25]	Multi-centre mixed methods: Surveys Interviews Focus groups	Assessment and delivery	8 health service organizations	Clinicians (n = 43) Clients (n = 228)	Assessment and delivery of OHL-interventions in three phases: - assessment using HLQ questionnaire to identify local strengths - needs and problems, results used by stakeholders to identify local solutions - local stakeholders prioritize action areas and co-design interventions - interventions implemented through quality improvement cycles Principles of the Ophelia approach: focused outcomes, equity driven, needs diagnosis, co-design, driven by local wisdom, sustainable, responsive, and systematically applied.
Goeman (2016) [26]	Mixed methods: Surveys Interviews	Assessment and delivery	Home nursing service setting (7 sites)	Nurses (n = 9) Clients with diabetes (n = 113)	Assessment, development and pilot of tailored diabetes self-management intervention: - education tool - online resources - teach-back training
Jessup (2018) [27]	Mixed methods: Surveys Interviews	Assessment and delivery	8 health service organizations	Staff (n = 23) Patients (n = 384)	Assessment and co-design of local OHL-interventions targeting: - patients - provider-patient interface - system-level
Cawthon (2014) [33]	Mixed methods: Observation Focus group Interviews Process recordings	Delivery	University medical centre	Nurses and staff (number not reported) Patients (n = 74,249)	Implementation of the three Brief Health Literacy Screening items into the nursing work flow: - How confident are you when filling out medical forms by yourself? - How often do you have someone help you read hospital materials? - How often do you have problems learning about your medical condition because of difficulty understanding written information? Implementation guided by a quality improvement framework consisting of leadership support, training, monitoring uptake of screening items, and feedback
Mabachi (2016) [28]	Qualitative study Interviews Observation	Assessment and delivery	Primary care practices (n = 12)	3 staff members per practice (total N = 36)	Assessment and delivery of 13 of the 20 tools in the HLUP Toolkit in one or more practices. Tools were organized under five sections: - path to improvement - improve spoken communication - improve written communication - improve self-management and empowerment - improve supportive systems
Brega (2015) [29]	Mixed method pre-post study Interviews Rating materials	Assessment and delivery	Primary care practices (n = 4)	Professionals (n = 12) 3 per practice	Assessment and delivery with HLUP toolkit 11: - design Easy-to-Read Material
Kaper (2019) [42]	Mixed methods: Surveys Interviews Observation	Assessment and delivery	Hospitals (n = 4)	Staff (n = 24) Older adults (n = 40)	- Assessment (using Quickscan Health literacy toolbox [in NL] and Literacy Audit for Health Care Settings [in IRL]), on four domains: navigation, digital-, written-, and oral communication - Planning and delivery of interventions to improve navigation and digital-, - written-, and oral communication
Vellar (2017) [30]	Mixed methods: Observation Interviews Survey	Assessment and delivery	Regional health service (9 hospitals)	Health professionals & various staff (exact number not reported) Patients (n = 1179)	Design of OHL-framework in three phases: 1. review of literature and clinical incidents; 2. organizational consultations; 3. piloting of HL strategiesFocus of OHL-framework: ensure effective communication, embed HL in health systems, and integrate HL into clinical incident management, education and clinical QI
Mastroianni (2019) [41]	Quantitative pre-post study: Rating of materials	Assessment and delivery	Regional health service (9 sites)	Sample: NA	Implementation of the PiP (Patient information Portal) process: - organization-wide approach for staff to develop plain-language patient information together with patients - supported by an interactive intranet site, a coordinator, and an HL ambassador training program
Weaver (2012) [31]	Mixed methods: Observation Interviews	Assessment	Clinics of a rural health centre (n = 3)	Various staff (n = 19) Patients (n = 16)	Assessment on six domains using an open-ended approach (informed by toolkits of: HLEHHC, Joint commission, the HLUP and AHRQ): - patient–provider interaction - patient education - printed materials - technology - inter-staff interaction - policy
Wray (2019) [32]	Qualitative study: Interviews	Delivery	Clinics of a rural health centre (n = 3)	Various staff (n = 19) Patients (n = 16)	Planning and delivery of interventions to enhance health literacy: - staff orientation to increase knowledge of HL and HL-friendly practices - formation of task force from several staff levels - development of a logic model and strategic planning of activities to enhance HL - improvement of complicated patient forms, and plain language diabetes self-care patient education materials - implementation of HL practices with staff at each level - identification of criteria for HL outcomes for program evaluation: increased HL awareness and capacities, HL practices, and sustainability in these practice

**Table 2:** Outcomes of OHL Assessments and Interventions.

Stage	Outcome Level		
	Patient	Professional	Organization
**OHL-Assessment**	**Problems with communication and navigation [3,17,23,25,28,29,30,31,34,36,37,38,39,40,41,42,43].** - Navigation: difficulties due to inconsistent terms and signage in larger buildings. - Written- and digital information too long and complex due to high reading levels. - Oral communication: difficulty with understanding information and participating in treatment. **Positive experiences [3,31,36,40]:** - Satisfaction on interaction with providers - Staff responsive to help with navigation, questions, and explaining information. - Information easy to read and accessible.	**OHLproblems identified among staff [3,17,24,25,26,27,30,31,34,35,36,37,38,39,40,42,45]:** - Limited awareness and knowledge of HL (difference between individual and OHL). - Lack of HL training. - Limited application of HL practices. **Positive experiences** [3,31,34,36,40,42]: - Patient-centred attitude and commitment to provide high quality care. - Awareness of HL issues and (self-reported) application of HL practices. - OHL-assessment reported to increase awareness and understanding of OHL barriers, especially assessment with patients.	**OHL problems identified across organizations [17,25,30,31,35,38,42,43]** - OHL not a strategic priority, although its importance is acknowledged. - Organizational cultures vary in fostering organizational change and quality improvement. - OHL policies and structures lacking; e.g., to improve patient centredness, empowerment, and comprehensible communication. - Lack of systematic routine procedures to address HL problems, coordination and delivery of care, community resources, and to engage patients.
**Delivery of OHL-interventions**	**Some positive patient outcomes after organization-wide OHL-interventions** [25,26,27,30,41]: - Small to greater improvement of individual HL levels after educational interventions. - Behaviour changes after intervention with community volunteers. - Some positive impact of patient–provider interventions. - Increased patient engagement/input on improving written health information and services.	**Positive intermediate outcomes on competency, communication, and practices after organization-wide OHL-interventions** [25,26,27,30,32,33,41,42]: - Greater commitment and competency to address health literacy and communication after training. - Increased application of health literacy practices. - Improved provider-patient interaction. **Intermediate outcomes on written communication** [29,32,41,42]: - Wider assessments and revision of materials - Positive, but varying, improvement regarding comprehensibility and actionability of materials.	**Positive intermediate organizational outcomes after organization-wide OHL-interventions** [25,26,27,30,32,33,41,42]: - Embedding of OHL into organizational processes as strategic priorities, frameworks, and policies. - Organization-wide platform to revise materials. - Redesign of service procedures to improve health literacy screening, access, and patient engagement. - Design of more comprehensible websites. - Staff capacity building on HL, comprehensible communication, and self-management. **Limited improvement reported** [25,28,29,32,42,43]: - Struggle to define priorities and action plans - Navigation and protocols on communication. - Sustainable and routine application of HL practices.

**Table 3:** Factors and strategies influencing assessment and delivery of OHL-interventions.

Stage	Outcome Level		
	Patient Level	Professional Level	Organizational Level
**OHL-assessment**	Facilitators [3,24,25,30,42] - Involving patients in assessment - Barriers [42] - Lack of patient-perspective - Effort to recruit patients	Facilitators [3,22,28,31,34,42,43]: - Introduction meetings to increase HL awareness and staff buy-in. - OHL–assessment perceived as relevant and feasible. - Tool features: adaptable, clear structure, feasible to use. - Staff commitment Barriers [28,34,37,38,39,42,43]: - Assessments perceived as lengthy and resource-intensive - Turnover and part-time working staff - Assessment requiring more time than anticipated - Limited knowledge of quality improvement	Facilitators [3,28,31,34,35,39,42,43]: - Comprehensive assessments - Assessments applied in stepwise and flexible manner. - Change champion and project-committees - Support from leaders and researchers. - Culture and strategies for quality improvement. Barriers [17,28,34,37,38,39,42,43]: - Limited resources. - Limited knowledge of quality improvement. - Variety in departments increases difficulty of HL assessment.
**Delivery of OHL-interventions**	Facilitator [25,26,27,30,41] - Patient engagement in evaluating information and health services. - Patients taking part in interventions to improve outcomes	Facilitators [25,26,27,30,32,33,41,42]: - Staff commitment - Staff involved in co-design of interventions, planning processes, and quality improvement cycles. - Staff meetings to discuss HL - Staff having knowledge of change strategies and quality improvement Barriers [28,29,42,43]: - Staff with limited knowledge of health literacy concept	Facilitators [25,26,27,30,32,33,41,42]: - Support from leaders and researchers. - Accountability. - Organization-wide approach: strategic and collaborative planning and development of program logic models combining top-down and bottom-up approaches. - Detailed, coordinated and concrete action plans - Co-design process to develop and pilot interventions - Quality improvement cycles to pilot test and refine interventions. - Practices affiliated with larger health systems Barriers [28,29,32,42,43]: - Limited leadership support - Limited resources - Lack of systematic approach to coordinate implementation. - Time required for implementation activities - Bureaucratic and technological barriers - Lack of coordination with other quality improvement initiatives - Restrictions related to navigation guidelines

## Supplementary Materials

The following are available online at https://www.mdpi.com/article/10.3390/ijerph182211906/s1, Table S1: Overview detailed search strategy.
