# Supplementary material for: Outcomes and Critical Factors for Successful Implementation of Organizational Health Literacy Interventions: A Scoping Review
Source: Int J Environ Res Public Health. 2021 Nov 12;18(22):11906. doi: 10.3390/ijerph182211906 (PMC8622809; doi:10.3390/ijerph182211906)
Supplement: Supplementary file 1 [file ijerph-18-11906-s001.zip › ijerph-1427927-supplementary.pdf]

S1. Overview detailed search strategy

Steps	Pubmed	Scopus	Cinahl	Psychinfo
**Step 1. Terms relating to "health literacy"**	health literacy[ti] OR health literate[ti] OR "Health Literacy/organization and administration"[Mesh] AND	TITLE "health literacy" OR "health literate" AND	MH "Health Literacy/OG" OR TI health literacy OR health literate AND	MM "Health Literacy" OR MM "Mental Health Literacy" OR TI health literacy OR health literate AND
**Step 2. Terms relating to "health care setting"**	"Community Health Services"[Mesh] OR healthcare system*[tiab] OR "Primary Health Care"[Mesh] OR primary care[tiab] OR "Delivery of Health Care/organization and administration"[Mesh] OR organization*[tiab] OR organisation*[tiab] OR hospital*[tiab] OR institution*[tiab]	TITLE-ABS KEY organization* OR orga nisation* OR"healthcare system*" OR "primary care" OR "primary health care" OR "primary healthcare" OR hospital*	MH "Primary Health Care" OR MH "Health Care Delivery+/AM" OR MH "Community Health Services" OR MH "Community Mental Health Services+" OR MH "Community Health Nursing+" OR MH "Home Health Care+" OR MH "Preventive Health Care+" OR TI (organization* OR organisation* OR healthcare system* OR primary care OR primary health care OR primary healthcare OR hospital* OR institution*	DE "Public Health Services" OR DE "Primary Health Care" OR TI (organization* OR organisation* OR healthcare system* OR primary care or primary health care or primary healthcare OR hospital* OR institution* OR AB organization* OR organisation* OR KW organization* OR organisation* OR healthcare system
**Step 3. Search terms relating to "Intervention characteris-tics"**	OR "Organizational culture"[Mesh] OR "Organizational Innovation"[Mesh] OR "Organization and Administration"[Mesh] OR "Program Development"[Mesh] OR "Professional-Patient Relations"[Mesh] OR "Culturally Competent Care"[Mesh] OR program development[tiab] OR professional patient relation*[tiab] OR cross	OR "program development" OR "professi onal patient relation*" OR "cross cultural communication" OR "interc ultural communication"	OR MH "Organizational Culture+" OR MH "Organizational Change" OR MH "Program Development+" OR MH "Professional-Patient Relations+" OR MH "Cultural Competence" OR program development OR professional patient relation* OR cross cultural communication OR intercultural communication	OR DE "Health Care Delivery" OR DE "Clinical Practice" OR DE "Health Care Access" OR DE "Health Care Costs" OR DE "Health Care Reform" OR DE "Health Care Utilization" OR DE "Managed Care" OR DE "Quality of Care" OR DE "Quality of Services" OR DE "Clinical Practice" OR DE "Case Conceptualization" OR DE "Health Care Access" OR DE "Treatment Barriers" OR DE "Utilization Reviews" OR DE "Health Maintenance Organizations" OR DE "Organizational Change" OR DE

	cultural communication[tiab] OR intercultural communication[tiab]			"Organizational Climate" OR DE "Innovation" OR DE "Program Development" OR DE "Therapeutic Processes" OR program development OR professional patient relation* OR cross cultural communication OR intercultural communication)
**Numbers retrieved per database**	**1511**	**1351**	**1750**	**808**
